# Spatiotemporal Role of Transforming Growth Factor Beta 2 in Developing and Mature Mouse Hindbrain Serotonergic Neurons

**DOI:** 10.3389/fncel.2019.00427

**Published:** 2019-09-20

**Authors:** Enaam Chleilat, Robert Mallmann, Rainer Spanagel, Norbert Klugbauer, Kerstin Krieglstein, Eleni Roussa

**Affiliations:** ^1^Institute of Anatomy and Cell Biology, Department of Molecular Embryology, Faculty of Medicine, Albert Ludwigs University Freiburg, Freiburg, Germany; ^2^Institute for Experimental and Clinical Pharmacology and Toxicology, Faculty of Medicine, Albert Ludwigs University Freiburg, Freiburg, Germany; ^3^Institute of Psychopharmacology, Central Institute of Mental Health (ZI), Heidelberg University, Mannheim, Germany

**Keywords:** raphe nucleus, serotonin, aminergic, early growth response 2, neurogenesis, neurochemistry, behaviour

## Abstract

Transforming growth factor betas are integral molecular components of the signalling cascades defining development and survival of several neuronal groups. Among TGF-β ligands, TGF-β2 has been considered as relatively more important during development. We have generated a conditional knockout mouse of the *Tgf-β2* gene with knock-in of an EGFP reporter and subsequently a mouse line with cell-type specific deletion of TGF-β2 ligand from Krox20 expressing cells (i.e., in cells from rhombomeres r3 and r5). We performed a phenotypic analysis of the hindbrain serotonergic system during development and in adulthood, determined the neurochemical profile in hindbrain and forebrain, and assessed behavioural performance of wild type and mutant mice. Mutant mice revealed significantly decreased number of caudal 5-HT neurons at embryonic day (E) 14, and impaired development of caudal dorsal raphe, median raphe, raphe magnus, and raphe obscurus neurons at E18, a phenotype that was largely restored and even overshot in dorsal raphe of mutant adult mice. Serotonin levels were decreased in hindbrain but significantly increased in cortex of adult mutant mice, though without any behavioural consequences. These results highlight differential and temporal dependency of developing and adult neurons on TGF-β2. The results also indicate TGF-β2 being directly or indirectly potent to modulate neurotransmitter synthesis and metabolism. The novel floxed TGF-β2 mouse model is a suitable tool for analysing the *in vivo* functions of TGF-β2 during development and in adulthood in many organs.

## Introduction

The raphe system of the hindbrain consists of nine brainstem nuclei, containing serotonin-producing neurons. Hindbrain serotonergic neurons innervate most regions of the brain and spinal cord and regulate many homeostatic and behavioural processes. Anatomically, hindbrain serotonergic neurons form two clusters, the rostral and caudal subgroups, each subpopulation revealing distinct molecular signatures. Many genes, among them those encoding for transcription factors, intracellular signalling, ion transport, and axon guidance are differentially enriched in rostral and caudal 5-HT subgroups (Wylie et al., 2010; Okaty et al., 2015). Moreover, development of elegant genetic tools has contributed to uncovering the molecular, cellular, and functional diversity within the hindbrain serotonergic system and even within individual raphe nuclei (Jensen et al., 2008; Alonso et al., 2013; Okaty et al., 2015; Teissier et al., 2015; Niederkofler et al., 2016). Hindbrain 5-HT subgroups derive from distinct rhombomeric sublineages: within the rostral group, DR exclusively derives from rhombomere (r) 1, whereas median raphe (MR) is populated by r1-, r2-, and r3-derived neurons. The main caudal raphe nucleus, the RM originates from r5 and r6, while RPA and RO are exclusively populated by r6-derived neurons (Jensen et al., 2008; Alonso et al., 2013).

Caudal hindbrain serotonergic neurons have originally been considered to provide innervation to the cerebellum and spinal cord. However, current knowledge emerged from recent studies has detected that r3/r5-derived neurons innervate the tegmental nuclei of Gudden, as well as the medullary nuclei, including locus coeruleus. More rostrally, axons deriving from r3/r5 were detected in the piriform cortex, the cortex of amygdala, and in cell layers of the dentate gyrus and hippocampal CA1 region. Importantly, r3/r5- axons provide innervation to other rostral and serotonergic neuron subgroups, such as to the DR (Bang et al., 2012; Alonso et al., 2013). Biologically relevant, but far from being completely understood, individual 5-HT neuron subtypes have been linked to specific physiological functions, as well as to cognitive and behavioural performances (Bang et al., 2012; Whitney et al., 2016). As an example, breathing reflex is specifically driven by Pet1-and Egr2-positive 5-HT neurons, the neurons functioning as P_CO__2_/pH chemoreceptor (Brust et al., 2014). Axons from these neurons have been detected in nuclei involved in respiratory control, as well as in pre-Bötzinger complex (Bang et al., 2012).

Egr2 (early growth response-2; also known as Krox20) is a zinc finger early-immediate transcription factor, whose expression in the developing brain is detected as early as E8 and is restricted to r3 and r5, where it regulates Hox genes required for hindbrain segmentation (Schneider-Maunoury et al., 1997; Labalette et al., 2015). Krox20 null mice reveal perinatal lethality (Topilko et al., 1994). During development, in Krox20 mutants, r3 cells will acquire r2 or r4 identity and r5 cells will be r6 (Voiculescu et al., 2001). Krox20 expression is induced in response to several stimuli, including injury-derived mechanical forces, cellular stress, cytokines, and growth factors in non-neuronal cellular paradigms. Intriguingly, phenotype analysis of the brainstem of *Tgf-β2^–/–^* mutants at embryonic day (E) 18 showed impaired synaptic transmission of spontaneous GABAergic/glycinergic and glutamatergic post-synaptic currents in the respiratory control area, the pre-Bötzinger complex (preBötC), and has been proposed as the likely cause of perinatal death in *Tgf-β2^–/–^* mutants (Heupel et al., 2008).

Although the physiological significance of individual TGF-β isoforms on the induction, differentiation, survival, and maintenance of caudal 5-HT subpopulations is not yet elucidated, we have previously shown that during development of midbrain dopaminergic neurons, TGF-β2 is relatively more important, compared to TGF-β3, since reduction in the number of TH-expressing cells in the ventral midbrain in *Tgf-β2^–/–^/Tgf-β3^+/–^* mutants was higher than in *Tgf-β2^+/–^/Tgf-β3^–/–^* mice (Roussa et al., 2006). In a recent study, we have demonstrated a selective growth factor dependency of individual rostral hindbrain serotonergic subpopulations. *Tgf-β2* null mutant mice revealed impaired development of rostral hindbrain serotonergic neurons at E12 and selective loss of PMR 5-HT neurons at E18 (Chleilat et al., 2018). Moreover, conditional deletion of the whole TGF-β signalling from rhombomere 1 leads to impaired development of dorsal raphe 5-HT neuron subgroups in a temporal manner (Chleilat et al., 2018). The putative effects of TGF-β ligands on the development of caudal hindbrain serotonergic neurons has not been addressed so far.

In the present study, we complement our previous studies to come full circle and examine the development of the caudal hindbrain serotonergic system. We have generated a conditional knockout mouse of the *Tgf-β2* gene with knock-in of an EGFP reporter and subsequently a mouse line with cell-type specific deletion of TGF-β2 ligand from Krox20 expressing cells (i.e., in cells from r3 and r5). We performed a phenotypic analysis of the hindbrain serotonergic system during development and in adulthood, determined the neurochemical profile in hindbrain, cortex, and hippocampus and assessed behavioural performance of WT and mutant mice.

## Materials and Methods

### Antibodies and Reagents

The following antibodies were used as primary antibodies: rabbit anti-5-HT (S5545) purchased from Sigma-Aldrich (Taufkirchen, Germany), anti-cleaved caspase 3 from Cell Signaling (Frankfurt, Germany, #9662 for western blots and, #9664/#9661 for immunofluorescence), anti-GFP (ab6556) and anti-Ki67 (ab16667) from Abcam (Cambridge, United Kingdom). Mouse monoclonal anti-GAPDH was from Abcam ([6C5], ab8245) and anti-β-III-tubulin was from Developmental Studies Hybridoma Bank (Iowa City, IA, United States). Additionally, goat anti-rabbit-biotin (111-065-144) or peroxidase-antiperoxidase complex of rabbit (R/PAP) and goat anti-rabbit [GAR/IgG (H + L)] were from Dianova. Vectastain ABC kit (Elite PK-6100 standard) and DAB peroxidase substrate kit (sk-4100) were from Biozol. Normal goat serum (#C07SA) and normal donkey serum (#C06SBZ) were from Bio-Rad (Puchheim, Germany).

### Animals

All protocols were carried out in accordance with German ethical guidelines for laboratory animals and approved by the Institutional Animal Care and Use Committee of the City of Freiburg and the University of Freiburg (authorizations: G11/56, G17/008, and X-16/07S). All mouse embryos used in this study were maintained on C57BL6/J background.

### LoxP-Flanked *Tgf-β2* Mice

A conditional knock-out of the *Tgf-β2* gene with knock-in of an EGFP reporter was generated. The *Tgfβ2^*flox/flox*^* mice carrying homozygous loxP site insertion flanking exon 1 of the *Tgf-β2* gene have been generated by Ozgene (Bentley, WA, Australia). TGF-β2 genomic sequence, located in chromosome 1, nucleotides 188420000–188550000, was retrieved from the Ensembl Mouse Genome Server^1^. Ensembl gene ID: ENSMUSG00000039239. The conditional knock-in strategy was such that WT sequence can be replaced by the reporter sequence via Cre-mediated inversion of a region flanked by lox66 and lox71 sites (Zhang and Lutz, 2002; Oberdoerffer et al., 2003).

**FIGURE 1:**
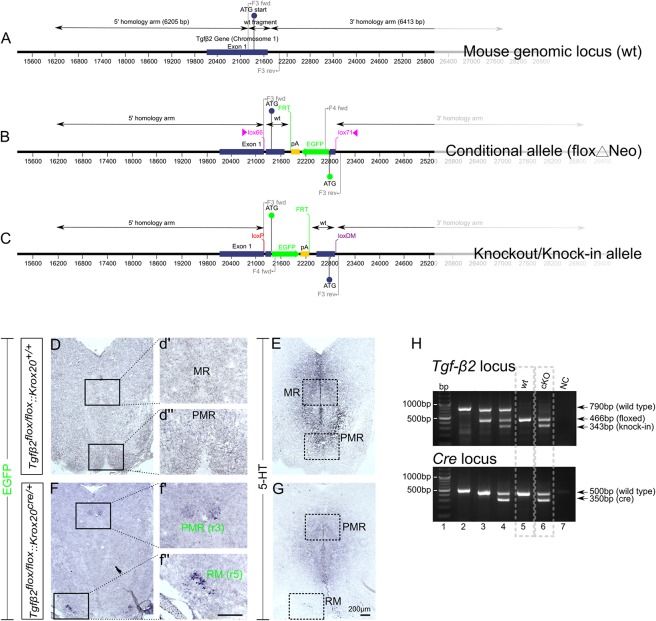
Conditional deletion of *Tgf-β2* in Krox20-expressing cells. **(A–C)** Schematic illustration of targeting strategy to generate the conditional deletion of *Tgf-*β*2*. Wild type *Tgf-β2* allele (WT), the conditionally deleted *Tgf-β2* allele (Δ) and the knockout/Knock-in allele (KI). **(D,F)** Immunoperoxidase labelling for EGFP in hindbrain of WT *Tgfβ2^*flox/flox*^:Krox20^+/+^* and *Tgfβ2^*flox/flox*^:Krox20^*cre*^*^/+^ mice at E18. PMR and RM are positive for EGFP in the mutants, but not in WT animals. (r): rhombomere. **(d′,d′′,f′,f′′)** Represent higher magnification of the respective black-boxed areas in **(D,F)**. **(E,G)** Immunoperoxidase labelling for 5-HT in hindbrain of *Tgfβ2^*flox/flox*^:Krox20^+/+^* and *Tgfβ2^*flox/flox*^:Krox20^*cre*^*^/+^ mice. Scale bar: 200 μm. **(H)** Representative genotyping results. *Tgf-β2* locus: lane 1: bp ladder, lane 2: two *Tgf-β2 wt* alleles, lane3: one *wt* and one “floxed” *Tgf-β2* allele, lane 4: one *wt Tgf-β2* allele and a partially recombined floxed allele, lane 5: two floxed *Tgf-β2* alleles, and lane 6: two recombined *Tgf-β2* floxed alleles, NC: negative control. Dotted boxes indicate the genotypes used for phenotypic analysis, *Tgfβ2^*flox/flox*^:Krox20^+/+^ (wt) and Tgfβ2^*flox/flox*^:Krox20^*cre*^*^/+^ (*cKO*).

The WT exon 1 fragment contains *Nhe*I and *Cla*I sites for cloning, *Bgl*II and *Eco*RV sites for genomic screening and a lox66 site. The EGFP fragment was generated from a fusion of two fragments. Fusion fragment 1 contains a portion of the 5′ untranslated region from exon 1 and fusion fragment 2 contains the EGFP coding sequence. The EGFP fragment contains *Cla*I and *Asc*I sites for cloning, *Kpn*I and *Mfe*I sites for genomic screening and a lox71 site. The region flanked by lox66/lox71 sites includes the coding sequence and splice donor of exon 1, in forward orientation, and a cassette consisting of the EGFP coding sequence and a polyA signal, in reverse orientation. The mouse genomic locus (wt), the conditional allele (floxΔNeo), and the knockout/knock-in allele is schematically presented in Figures 1A–C.

Krox20-cre mice were provided by Dr. Carmen Birchmeier (Max-Delbrück-Center for Molecular Medicine, Berlin; Garratt et al., 2000). Mice with two *Tgf-β2^*flox/flox*^* alleles and one Krox20^Cre^ allele were crossbred to yield littermate matched *Tgfβ2^*flox/flox*^:Krox20^+/+^* (*wt*) and *Tgfβ2^*flox/flox*^:Krox20^*cre/+*^* (*cKO*) mice.

#### Genotyping

Genotyping was performed using RedTaq Mastermix from Genaxxon (Ulm, Germany). For a reaction, we used RedTaq Mastermix, DMSO and primers together with genomic DNA.

As indicated in Figures 1A–C, following primers were used: F3 primer pair : 5′ GGGCATTAACTTTCGACTGC 3′ (F3 forward) and 5′ ACCAGGGGAGAGGAGAAATG 3′ (F3 reverse): These primers allow the amplification of a 790 bp fragment from the WT allele.

F4 primer pair : 5′ GATGAACTTCAGGGTCAGCTTG 3′ (F4 forward) and 5′ ACCAGGGGAGAGGAGAAATG 3′ (F3 reverse): These primers allow the amplification of a 466 bp fragment from the flox alleles.

F5 primer pair: 5′ GGGCATTAACTTTCGACTGC (F3 forward) and 5′ GATGAACTTCAGGGTCAGCTTG (F4 forward): These primers allow the amplification of a 343 bp fragment from the KI alleles.

Multiplex PCR was performed with following cycle conditions: denaturation at 93°C for 3 min, and 35 cycles of PCR amplification at 93°C for 30 s and 62°C for 1 min and elongation at 72°C for 1:30 min were followed by 72°C for 10 min. PCR products were run on a 2% agarose gel in TAE buffer at 100 V, and then photodocumented using a UV transiluminator.

For amplification of Krox20 and Cre, the following primers were used:

(Krox20 forward): 5′-CACTACACCAGCAACTCCTGGCTCC-3′, (Krox20 reverse): 5′-CCCACCCACAAGCTCCGAAGAA-3′, (Cre-reverse): 5′-ATGCTCAGAAAACGCCTGGCGATCC-3′. The expected band for the *wt* gene is ∼500 bp, while the expected band for the Cre gene is ∼350 bp. PCR was performed with the following cycle conditions: denaturation at 93°C for 3 min, and 36 cycles of PCR amplification at 93°C for 45 s and 65°C for 2 min and elongation at 72°C for 2 min were followed by 72°C for 5 min. PCR products were run on a 2% agarose gel in TAE buffer at 100 V, and then photodocumented using a UV transiluminator.

#### Immunohistochemistry

Immunohistochemistry and immunofluorescence have been performed as described earlier (Roussa et al., 2006). Rabbit polyclonal anti-5-HT (1:1000), anti-cleaved caspase 3 (1:200), anti Ki67 (1:100) and anti-GFP (1:200), were used as primary antibodies. Goat anti- rabbit-biotin or anti-rabbit IgG and subsequently R/PAP, were used as secondary antibodies for 5-HT and GFP. Goat anti-rabbit Alexa 488 or Alexa 594 were used as secondary antibodies for immunofluorescence.

The numbers of 5-HT-labelled neurons were counted on the complete series of 10 μm transverse sections after immunoperoxidase. A neuron was designated as 5-HT positive if it revealed a darkly labelled cytoplasm and a clearly visible, unstained nucleus. Only cells fulfiling these criteria were included in the cell counts. To avoid double counting the same cell on two sequential sections, only every fourth section was counted.

#### *In situ* Hybridization

Non-radioactive *in situ* hybridization (ISH) on cryosections and preparation of digoxigenin-labelled probes were carried out as described by Ernsberger et al., 1995. Riboprobes were labelled with digoxigenin labelling kit (Roche, Mannheim, Germany) and revealed by BCIP/NBT (Roche). *Pet1* (Hendricks et al., 1999), *Gata2* and *Gata3* (Haugas et al., 2016) probes were kindly provided by Dr. Juha Partanen (Department of Biosciences, University of Helsinki, Helsinki, Finland), and the *Neurofilament* ISH probe was provided by Dr. Katrin Huber (Department of Medicine, University of Fribourg, Fribourg, Switzerland; Huber et al., 2002).

Images from *Pet1* ISH in *wt* and *cKO* were acquired with a ZEISS Imager M2 microscope, equipped with a AxioCam HRc camera. The Pet1-positive region was demarcated and ImageJ (NIH) was used to measure the *Pet1* ISH area of rostral and caudal raphe nuclei. After quantification data were normalised to the mean of *wt*.

#### Immunoblotting

For isolating proteins from hindbrain of 4%-PFA fixed brain cryosections from WT and *cko*, the method described by Scicchitano et al. (2009) has been applied, using the Qproteome FFPE Tissue Kit from Qiagen, following the manufacturer’s instructions. After determination of protein concentration, electrophoresis and blotting procedures were performed as described (Osterberg et al., 2011). Primary antibodies were diluted: anti-cleaved caspase 3 1:1000, anti GAPDH 1:10,000, anti-β-III tubulin 1:1000 Blots were developed in enhanced chemiluminescence reagents and signals were visualised on X-ray film. Subsequently, films were scanned and the signal ratio protein of interest: GAPDH, was quantified densitometrically. Differences in signal ratio were tested for significance using Student’s *t*-test. Results with levels of ^∗^*p* < 0.05 were considered significant.

#### Neurochemical Analysis of Tissue Punches From Adult *Tgfβ2^*flox/flox*^:Krox20^*cre/+*^* Mice and Electrochemical Detection

Neurochemical analysis of tissue punches from adult *Tgfβ2^*flox/flox*^:Krox20^*cre/+*^* mice and subsequent HPLC were performed as previously described (Vengeliene et al., 2017). Briefly, adult WT and cKO mice were sacrificed by cervical dislocation, and brains were quickly removed and freezed in liquid N_2_. Brains were then wrapped with aluminium foil and stored at −80°C until analysis. Brains were sliced in coronal sections of 120 μm width. Different regions were extracted by punching with a set of needles of several diameters ranging from 0.5 to 1.0 mm (FMI, Seeheim-Jugenheim, Germany) and collected into vials. The identification of regions was based on landmarks from the stereotaxical descriptions of The Mouse Brain Atlas (Paxinos and Watson, 1998). The following brain sites were collected and stored in −80°C: hindbrain, hippocampus, and cortex.

For HPLC analysis tissue samples were thawed, weighted and immediately homogenised in an extraction solution (0.1M perchloric acid, 1 mM EDTA) using a tissue homogeniser Mixer Mill (Qiagen, Hilden, Germany). Subsequently, obtained homogenates were cleared by centrifugation at 15000 *g* for 10 min at 4°C and supernatants analysed by an HPLC system, which consisted of a Spark Triathlon autosampler (Spark Holland B.V., Emmen, Netherlands), an Antec Leyden LC-100 pump (Antec Leyden, Zoeterwoude, Netherlands), a 150 mm × 2.0 mm C18-OptiAqua reverse phase column (3 μm particle size; VDSOptilab, Berlin, Germany) and a Decade II electrochemical detector (Antec Leyden, Zoeterwoude, Netherlands). The mobile phase was 50 mM sodium citrate, 2.4 mM sodium octyl sulfate, 0.1 mM EDTA, 10 mM NaCl, and 22% methanol at pH 4.0. The temperature applied on the system was 37°C. Tissue concentrations were determined by normalising the quantified amounts of the respective neurotransmitter or the metabolite to the corresponding weight of the individual tissue sample.

#### Behavioural Studies

Animals were housed in a temperature, humidity controlled vivarium with a 12 h light–dark cycle, food, and water were available *ad libitum*. To exclude possible influences of complex environmental enrichment on behaviour, only nest-building material was available to the animals (van Praag et al., 2000). Behavioural experiments were performed with adult (12–14 weeks old) male mice only. All behavioural phenotyping tests were performed during second half of the day cycle. Prior to each behavioural test, mice were transported in their home cages to the experimental room and allowed to acclimate for at least 1 h. One cohort of mice was used to perform the elevated plus maze and open field test at two consecutive days. Activity and behaviour of mice were observed using an automatic video tracking system for recording and analysis (VideoMot2 system V6.01, TSE, Bad Homburg, Germany). Another cohort was used for the forced swimming test. An experimenter who was blind to the genotype of the animals performed all experiments. The open field test and elevated plus maze were performed as described in detail in Mallmann et al. (2013).

##### Open field

The open field consisted of a square of 50 cm × 50 cm surrounded by a 35 cm opaque wall. The behaviour in the open field was recorded for 20 min. Evaluation of data sets included time spent in the central area, covered distance at central area and total covered distance.

##### Elevated plus maze

The elevated plus maze device consisted of two open and two closed arms each of 30 cm × 5 cm, closed arms were surrounded by 15 cm high opaque walls. All arms emerged from a central platform and were elevated 45 cm above the floor. Covered distance and duration of stay of the mice on each arm were continuously recorded during 7.5 min.

##### Forced swimming test

A cylindrical glass tank (30 cm height x 20 cm diameter) was used for the mouse forced swim test (FST). The water level was adjusted to 15 cm from the bottom. The temperature of the water was 24 ± 1°C. Mice were carefully placed in the water tank and behaviour was video recorded for 6 min. Only the last 4 min of each experiment were analysed. Mobility in the FST was defined as “any movements other than those necessary to balance the body and keep the head above the water” (Can et al., 2012).

### Statistical Analysis

Statistical tests were performed as indicated in the text. All tests were performed in GraphPad Prism, Version 7.04 for Windows. Data were tested for normality using a Shapiro–Wilk test and subsequently assessed for homogeneity of variance. If the data passed both tests, further analyses have been performed using the two-tailed unpaired Student’s *t*-test. For datasets with unequal variances, Welch’s correction was applied after Student’s *t-*tests. Values are reported as mean ± SEM, unless otherwise indicated. For datasets with non-normal distributions Mann–Whitney Rank Sum Test was used. For all statistical tests, *p* < 0.05 was considered statistically significant and *p-*values are indicated in the figures as follows: ^∗^*p* < 0.05, ^∗∗^*p* < 0.01, ^∗∗∗^*p* < 0.001, and ^∗∗∗∗^*p* < 0.0001.

## Results

As an approach to circumvent embryonic lethality of *TGF-β2-*mutant mice and to generate region and cell type specific deletions of *TGF-β2*, we have generated a conditional knockout of the *Tgf-β2* gene with knock-in of an EGFP reporter. The targeting strategy is illustrated in Figures 1A–C. The design of a conditional knock-in strategy was chosen, in which the WT sequence was replaced by the reporter sequence via Cre-mediated inversion of a region flanked by lox66 and lox71 sites, according to Zhang and Lutz (2002) and Oberdoerffer et al. (2003). Lox66 and lox71 sites flank the coding sequence between exon 1 and the EGFP cassette. The lox66 site is placed upstream of the start codon in the 5′ untranslated region of exon 1 and the lox 71 site is placed downstream of exon 1 within the first intron. Splicing from exon 1 to exon 2 occurs normally as there are no known splicing signals associated with the EGFP cassette. Cre-mediated recombination between the lox66 and lox71 sites results in the generation of a loxP site and a loxDM (double mutant) site (Figure 1C). Transcription starts at exon 1 but is terminated by the polyA signal in the EGFP cassette without splicing the downstream exons. Translation starts at the initiating ATG of the EGFP coding sequence. *Tgfβ2^*flox/flox*^:Krox20^*cre/+*^* mice lacking *Tgf-β2* in Krox20-expressing cells, i.e., in cells from rhombomere 3 (r3) and rhombomere 5 (r5), were generated by breading mice with two *Tgfβ2^*flox/flox*^* alleles with that with one *Krox20*^*Cre*^ allele.

According to the targeting strategy, in the conditional knockout animals, cells expressing Krox20 should express EGFP and lack expression of *Tgf-β2*. As shown in Figures 1D,F, in the *wt* (Figures 1D,d′,d′′) cells of the PMR and median raphe (MR) were devoid of EGFP immunoreactivity. In the cKO however (Figures 1F,f′,f′′) EGFP immunoreactivity was detectable in part of the PMR (presumably the r3-derived part) and of the r5-derived RM. Consecutive sections labelled for 5-HT (Figures 1E,G) showed considerable reduced number of immunopositive cells. *WT* and *cKO* were then processed for phenotypic characterization of the serotonergic system.

Figure 1H illustrates representative genotyping results using multiplex PCR with the primers described in *Material and Methods* and tailcuts as samples for genotyping. Figure 1H (upper panel) illustrates genotyping results of individual animals for the TGF-β2 locus only. With regard to *Tgf-β2* locus, one single band at 790 bp indicates two TGF-β2 WT- alleles (lane 2), the bands at 790 and 466 bp indicate one WT and one “floxed” TGF-β2 allele (lane 3). At lane 4, the band at 790 bp indicates one TGF-β2 WT allele. The two bands at 466 and 343 bp represent a partially recombined floxed allele. This partial recombination is due to the fact, that tail cuts were used as samples for genotyping. These tissue samples consist of several cell types, among them Krox20-posiitve Schwann cells (first described by Topilko et al., 1994). The Cre-recombinase activity in Krox20 positive Schwann cells is the reason for the presence of the knock-in band at 343 bp. One single band at 466 bp indicates two floxed TGF-β2 alleles (lane 5) and at lane 6, both floxed TGF-β2 alleles were partially recombined.

### Phenotypic Characterization of the Serotonergic System of *Tgfβ2^*flox/flox*^:Krox20^*cre/+*^* Mice During Embryonic Development

As the first step to investigate the involvement of TGF-β2 in the differentiation of r3 and r5-derived 5-HT neurons, we have phenotypically characterised mouse embryos at different developmental stages, namely at E12, E14, and E18. Since rostral and caudal 5-HT neurons consist of subgroups, we have determined, whenever possible to distinguish, the number of 5-HT neurons of the DR, caudal part of the dorsal raphe (DRc), MR, and PMR, representative for the subgroups within the rostral hindbrain 5-HT neurons, and of the caudal subgroups RM, raphe pallidus (RP), and raphe obscurus (RO) in *Tgfβ2^*flox/flox*^:Krox20^+/+^ (wt*) and *Tgfβ2^*flox/flox*^:Krox20^*cre/+*^* (*cKO*) mice.

**FIGURE 2:**
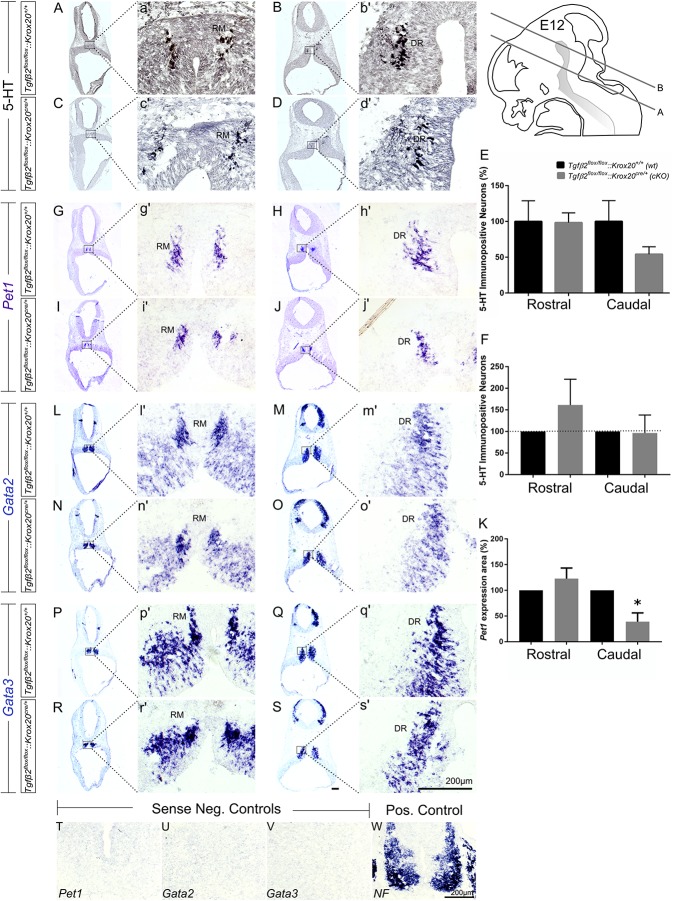
Phenotype analysis of *Tgfβ2^*flox/flox*^:Krox20^*cre/+*^* hindbrain at embryonic day 12 (E12). Immunoperoxidase light microscopy for rostral DR **(B,D)** and caudal RM **(A,C)** serotonergic neurons (5-HT positive) of *Tgfβ2^*flox/flox*^:Krox20^+/+^ (wt)* and *Tgfβ2^*flox/flox*^:Krox20^*cre/+*^ (cKO)* at E12. **(a′–d′)** Represent higher magnification of the respective black-boxed areas in **(A–D)**. **(E,F)** Counting of 5-HT positive cells after immunostaining in hindbrain tissue sections revealed no differences in the number hindbrain rostral and caudal serotonergic neurons between wild type (*wt*) and *Tgfβ2^*flox/flox*^:Krox20^*cre/+*^*. Not significant, using the two-tailed unpaired Student’s *t*-test (caudal) and Mann–Whitney Rank Sum Test (rostral), *n* = 5–7. Data are given as mean ± SEM, the mean of *wt* was set to 100 **(E)** or the mean of littermate was set to 100 **(F)**. ISH for expression of the serotonergic marker *Pet1* **(G–J,g′–j′)** using antisense probe on coronal fixed tissue cryosections from *wt* and *Tgfβ2^*flox/flox*^:Krox20^*cre/+*^* mouse embryos. **(K)** *Pet1* expression area in *cKO* was significantly decreased in the caudal hindbrain (^∗^*p* < 0.05, using the two-tailed unpaired Student’s *t*-test, *n* = 3). Data are given as mean ± SEM, the mean of *wt* littermate was set to 100. ISH for the early serotonergic markers *Gata2* **(L–O,l′–o′)** and *Gata3* **(P–S,p′–s′)**. **(T–W)** Represent the negative and positive controls for **(G–S)**. *NF*, neurofilament. Scale bar: 200 μm.

As shown in Figure 2, in *Tgfβ2^*flox/flox*^:Krox20^*cre/+*^* mice, at E12 (Figures 2C,c′), the total number of caudal hindbrain 5-HT immunopositive neurons, as represented by the neurons of RM (Figure 2E, 54.21 ± 10.35), was not significantly different from that of *wt* (Figures 2A,a′,E; 100.00 ± 29.01, *p* = 0.95, *n* = 5, cKO: *n* = 7). Similarly, the total number of rostral 5-HT neurons, as represented by the neurons of DR was also comparable between *cKO* (Figures 2D,d′,E 98.36 ± 13.58%) and *wt* (Figures 2B,b′,E 100 ± 28.85%, Mann–Whitney *U* = 17, *p* = 0.95, WT: *n* = 5, cKO: *n* = 7). When the neuronal counts of 5-HT neurons in *cKO* were normalised to the mean of *wt* littermate (Figure 2F), comparable values for rostral (161.3 ± 59.75) and caudal 5-HT (96.56 ± 41.65) neurons were assessed. Subsequently, ISH was performed for the transcription factors *Pet1* (Figures 2G–J), *Gata2* (Figures 2L–O), and *Gata3* (Figures 2P–S). *Pet1* is exclusively expressed in 5-HT neurons and therefore considered as a specific marker for 5-HT neurons (Hendricks et al., 1999). Interestingly, quantification of the *Pet1* expression area revealed significant decreased *Pet1* expression area in the caudal hindbrain of *cKO* (Figure 2K, 38.96 ± 17.12%, ^∗^*p* < 0.05, unpaired Student’s *t*-test, *n* = 3), compared to *wt*. In contrast, in the rostral hindbrain, no changes in *Pet1* expression area could be assessed (Figure 2K, 122.7 ± 20.7%). *Gata2* and *Gata3* are expressed in post-mitotic serotonergic and glutamatergic precursors. Whereas Gata2 is necessary for activation of serotonergic neuron-specific gene expression in the dorsal raphe, Gata3 is required for the expression of Tph2 and Sert (Haugas et al., 2016). Consistent with the 5HT immunolabelling results, *Gata2* (Figures 2L–O,l′–o′) and *Gata3* (Figures 2P–S,p′–s′) expression revealed similar distribution pattern and labelling intensity for RM and DR in *wt* and *cKO.* Labelling was considered specific, since incubation of the sections with the respective sense probes (Figures 2T–V) revealed no labelling. In contrast, ISH for *Neurofilament (NF)* (Figure 2W), used as positive control for ISH, revealed strong labelling intensity.

**FIGURE 3:**
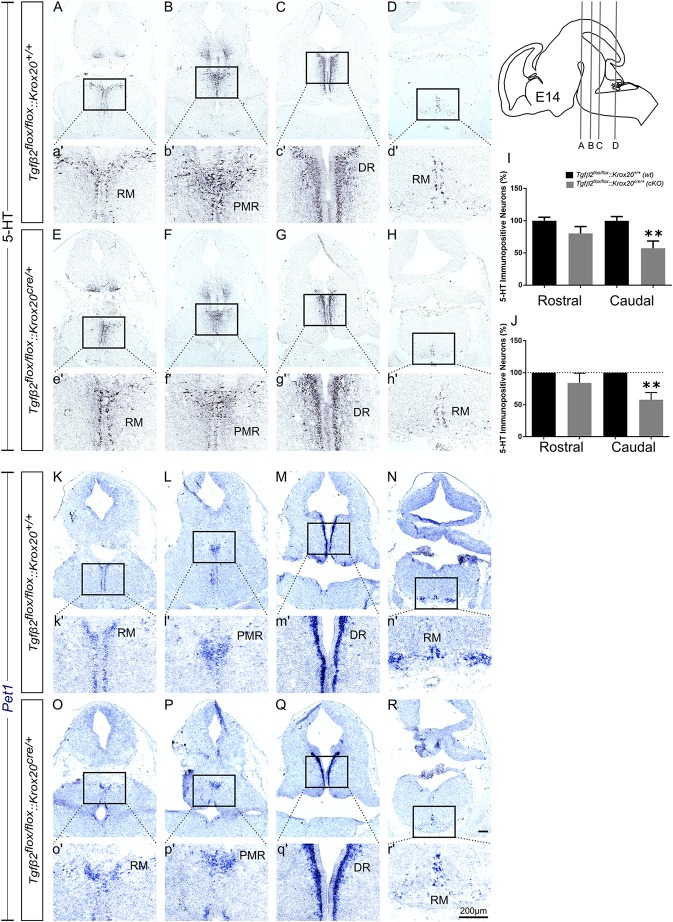
Phenotype analysis of *Tgfβ2^*flox/flox*^:Krox20^*cre/+*^* hindbrain at embryonic day 14 (E14). Immunoperoxidase light microscopy for the rostral serotonergic neuron subgroups DR **(C,G)**, and PMR **(B,F)** and of the caudal serotonergic neurons of RM **(A,E,D,H)** of *Tgfβ2^*flox/flox*^:Krox20^+/+^ (wt)* and *Tgfβ2^*flox/flox*^:Krox20^*cre/+*^ (cKO)* at E14. **(a′–h′**) represent higher magnification of the respective black-boxed areas in A-H. 5-HT positive cell counts **(I,J)** revealed significant decrease in the number of caudal hindbrain serotonergic neurons of RM between *Tgfβ2^*flox/flox*^:Krox20^*cre/+*^*, and wild type (*wt*) (^∗∗^*p* < 0.01, using the unpaired two-tailed Student’s *t*-test *n* = 6). Data are given as mean ± SEM, normalised to the mean of counts in *wt* set to 100 **(I)** or to the mean of *wt* littermate set to 100 **(J)**. ISH for expression of the serotonergic marker *Pet1* **(K–R**) using antisense probes on coronal fixed tissue cryosections from *wt* and *Tgfβ2^*flox/flox*^:Krox20^*cre/+*^*. **(k′–r′)** Represent higher magnification of the respective black-boxed areas in **(K–R)**. Scale bar: 200 μm.

At E14, as shown in Figure 3, the total number of caudal RM hindbrain 5-HT immunopositive neurons in *cKO* was significantly decreased (Figures 3E,H,e′,h′,I, 57.36 ± 11.08%), compared to *wt* (Figures 3A,D,a′,d′,I; 100.00 ± 6.5%; ^∗∗^*p* < 0.01, *n* = 6), whereas the rostral 5-HT neuronal subpopulations of DR (Figures 3C,G,c′,g′), and PMR (Figures 3B,F,b′,f′) exhibited comparable number of 5-HT neurons (Figure 3I, 100 ± 5.42 and 80.27 ± 10.66% for *cKO* and *wt*, respectively, *p* = 0.49, *n* = 6). Similar results were also obtained when the neuronal counts of 5-HT neurons of *cKO* were normalised to the mean of *wt* littermate. The number of rostral 5-HT neurons in *cKO* was 84.11 ± 15.14% of *wt*, whereas caudal hindbrain neurons in the *cKO* were significantly decreased compared to *wt* [57.77 ± 11.35%; ^∗∗^*p* < 0.01, *n* = 6 (Figure 3J)]. Accordingly, *Pet1* expression in RM was considerably weaker in *cKO* (Figures 3O,R,o′,r′), compared to *wt* (Figures 3K,N,k′,n′). Again, in line with the immunohistochemical results, *Pet1* expression in DR and PMR showed no obvious differences between *cKO* (Figures 3P,Q,p′,q′) and *wt* (Figures 3L,M,l′,m′).

**FIGURE 4:**
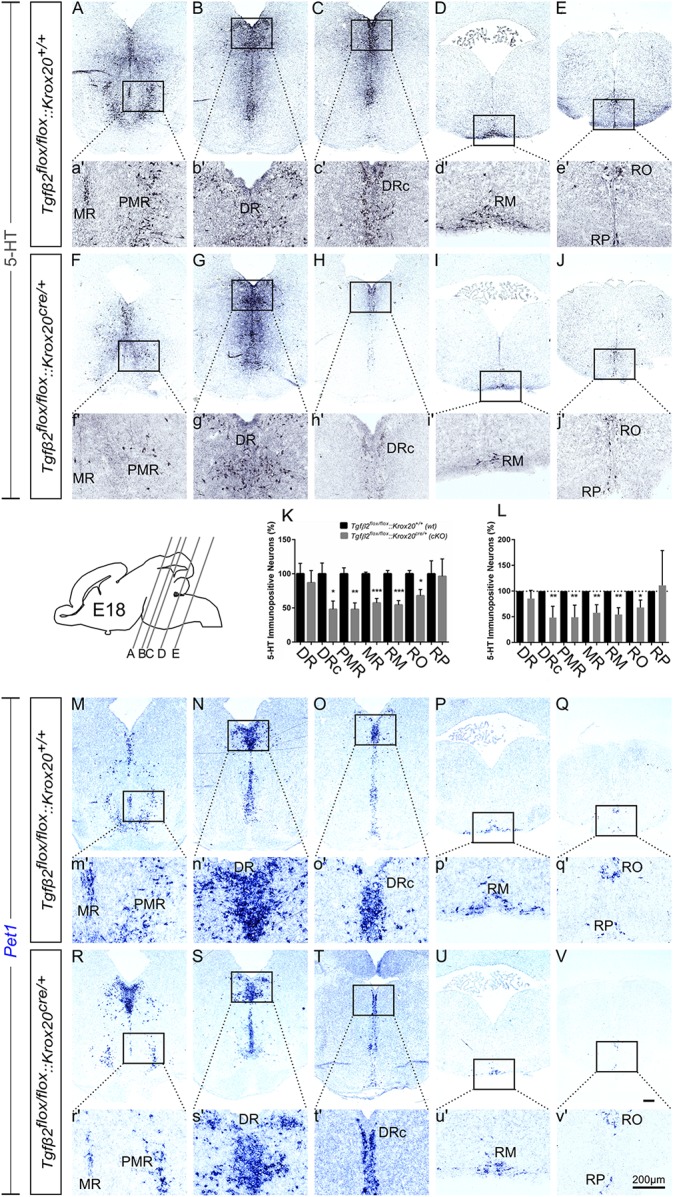
Phenotype analysis of *Tgfβ2^*flox/flox*^:Krox20^*cre/+*^* hindbrain at embryonic day 18 (E18). Immunoperoxidase light microscopy for the rostral serotonergic neuron subgroups (5-HT positive) DR **(B,G)**, caudal part of the dorsal raphe (DRc, **C,H**), median raphe (MR, **A,F**), and PMR **(A,F)**, and of the caudal serotonergic neuron subgroups RM **(D,I)** and RO **(E,J)** of *Tgfβ2^*flox/flox*^:Krox20^+/+^ (wt)* and *Tgfβ2^*flox/flox*^:Krox20^*cre/+*^* at E18. **(a′–j′)** Represent higher magnification of the respective black-boxed areas in **(A–J)**. Counting of 5-HT-positive **(K,L)** cells after immunostaining in hindbrain fixed tissue sections revealed significant decrease in the number of DRc, PMR, MR, RM, and RO but not of DR nor RP serotonergic neuron subpopulations between in *Tgfβ2^*flox/flox*^:Krox20^*cre/+*^* mutants, and wild type (*wt*) (^∗^*p* < 0.05 and ^∗∗^*p* < 0.01, using the unpaired two-tailed Student’s *t*-test *n* = 3–5). Data are given as mean ± SEM, the mean of *wt* was set to 100 **(K)** or the mean of *wt* littermate was set to 100 **(L)**. **(M–V)** ISH for expression of the serotonergic marker *Pet1*. Lower case images **(m′–v′)** represent magnification of the black-boxed area of the respective upper case images. Scale bar: 200 μm.

At E18, immunolabelling for 5-HT on coronal fixed cryosections revealed that the number of 5-HT immunopositive neurons was significantly decreased in the caudal part of DR (DRc; Figures 4C,H,K and higher magnification of the black-boxed area 4c′,h′, 48.05 ± 11.99%, ^∗^*p* < 0.05), in PMR (Figures 4A,F,K, higher magnification of the black-boxed area 4a′,f′, 47.89 ± 9.39%, ^∗∗^*p* < 0.01), in MR (Figures 4A,a′,F,f′,K 57.37 ± 6.45%, Mann–Whitney *U* = 0, ^∗∗^*p* = 0.007), and in the caudal RM (Figures 4D,I and higher magnification of the black-boxed area in 4d′,i′; 54.48 ± 6.17%; Mann–Whitney *U* = 0, ^∗^*p* < 0.01, *n* = 4) and RO (Figures 4E,e′,J,j′,K, 67.97 ± 9.02%, ^∗^*p* < 0.05) of the mutants, compared to *wt.* When the counts of 5-HT immunopositive cells in the individual subpopulations in the *cKO* animals were normalised to those of the respective *wt* littermate, as shown in Figure 4L, the significant decrease was also evident (48.45 ± 9.84% for DRc, ^∗∗^*p* < 0.01, Welch-corrected unpaired *t*-test, 49.08 ± 10.48% for PMR, ^∗∗^*p* < 0.01, Welch-corrected, unpaired *t*-test, 57.72 ± 7.02% for MR, ^∗∗^*p* < 0.01, Welch-corrected unpaired *t-*test, 54.48 ± 6.53% for RM, ^∗∗^*p* < 0.01, Welch-corrected unpaired *t*-test, 67.91 ± 8.43% for RO, ^∗^*p* < 0.05, unpaired *t*-test), compared to *wt*. Only the rostral subpopulation of the DR (86.97 ± 17.70 and 85.35 ± 7.19%) and of raphe pallidus (RP) (96.25 ± 25.45 and 111.1 ± 39.18%) exhibited comparable numbers of 5-HT immunopositive neurons in *wt* and *cKO*. These data have been further confirmed by ISH with the serotonergic marker *Pet1* (Figures 4M–V and magnification of the respective black-boxed areas).

### Phenotypic Characterization of the Serotonergic System of *Tgfβ2^*flox/flox*^:Krox20^*cre/+*^* Adult Mice

**FIGURE 5:**
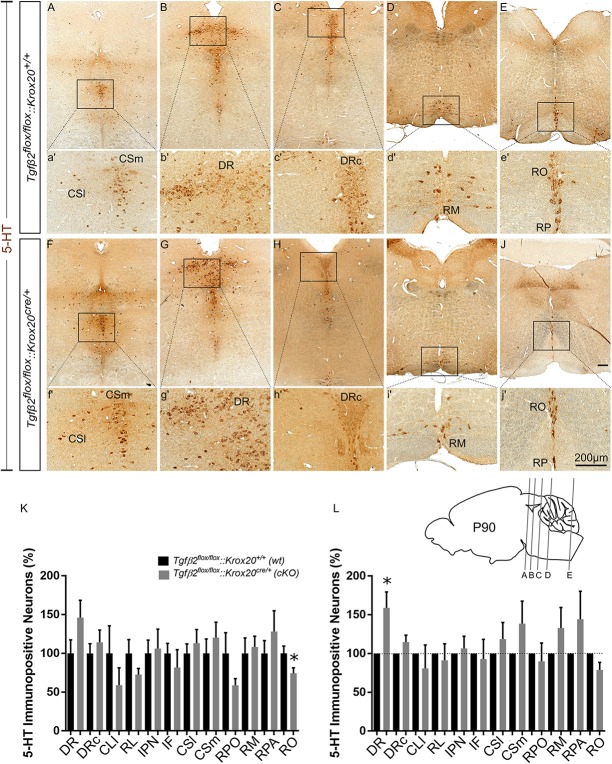
Phenotype analysis of *Tgfβ2^*flox/flox*^:Krox20^*cre/+*^* hindbrain in adult mice. Immunoperoxidase light microscopy for the serotonergic neuron subgroups (5-HT positive) DR **(B,G)**, caudal part of the dorsal raphe (DRc, **C,H**), superior central linear nucleus lateral part (CSl, **A,F**), superior central linear nucleus medial part (CSm, **A,F**) and of the caudal serotonergic neuron subgroups RM **(D,I)** RO **(E,J)** and raphe pallidus (RPA, **E,J**) of *Tgfβ2^*flox/flox*^:Krox20^*cre/+*^* in 3 month old mice. **(a′–j′)** Represent higher magnification of the respective black-boxed areas in **(A–J)**. Counting of 5-HT-positive **(K,L)** cells after immunostaining in hindbrain fixed tissue sections, ^∗^*p* < 0.05, using the Welch corrected unpaired or unpaired two-tailed Student’s *t*-test *n* = 5–8. Data are given as mean ± SEM, the mean of *wt* was set to 100 **(K)** or the mean of *wt* littermate was set to 100 **(L)**. Scale bar: 200 μm.

*cKO* animals are vital and fertile. As a next step we have phenotypically characterised adult, i.e., 3 month old animals, by using 5-HT immunohistochemistry. As shown in Figure 5K, in many subpopulations in adult *cKO*, the number of 5-HT immunopositive cells revealed a tendency to be higher than the *wt*, but these data did not reveal statistical significance (Figure 5K). Specifically, the number of 5-HT immunopositive cells in *cKO* was 146.2 ± 22.19% for DR (Figures 5B,b′,G,g′), *p* = 0.12, and 114.2 ± 15.92% for DRc (Figures 5C,c′,H,h′), *p* = 0.49, compared to the *wt*. Similarly, the 5-HT neurons of the superior central nucleus raphe, lateral part (CSl; that is the correlate of PMR in adults, Figures 5A,a′,F,f′) were 113.2 ± 17,63%, *p* = 0.55 and of the superior central nucleus raphe, medial part (CSm; the correlate of the median raphe in adults; Figures 5A,a′,F,f′) 120.3 ± 19.99%, *p* = 0.46, thus comparable between *wt* and *cKO*. Moreover, the number of 5-HT positive cells in the midbrain-located raphe nuclei, i.e., IF, IPN, RL, and CLI and of the RPO revealed no significance differences between *cKO* and *wt* (81.82 ± 22.88%, *p* = 0.50, 106.3 ± 25%, *p* = 0.84, 72.73 ± 7.82%, *p* = 0.18, 58.97 ± 22.4%, *p* = 0.34 and 58.82 ± 8.65%, *p* = 0.14, for IF, IPN, RL, CLI, and RPO, respectively). Only, the 5-HT neurons of the nucleus RO (Figures 5J,j′,E,e′) in *cKO*, a caudal 5-HT subpopulation located in the medulla, revealed significant decreased numbers of neurons (74.42 ± 6.99%, ^∗^*p* < 0.05), whereas other caudal subgroups, such as the RM (Figures 5D,d′,I,i′) and the nucleus raphe pallidus (RPA; Figures 5E,e′,J,j′) were comparable between *cKO* and *wt* (108.2 ± 13.78%, *p* = 0.73 and 128.1 ± 26.86%, *p* = 0.46, for RM and RPA, respectively).

When the counts of 5-HT immunopositive cells in the individual subpopulations in the *cKO* animals were normalised to those of the respective *wt* littermate, as shown in Figure 5L, a significant increase in the number of DR neurons was observed in the *cKO* (159 ± 20.47%, Welsh-corrected unpaired *t*-test, ^∗^*p* < 0.05, *n* = 8), compared to *wt*. In contrast, in the *cKO*, the number of 5-HT immunopositive cells in DRc (114.7 ± 8.84%, Welch-corrected unpaired *t*-test, *p* = 0.14), CSl (118.6 ± 21.4%, Welsh-corrected unpaired *t*-test, *p* = 0.41, *n* = 7), CSm (138.7 ± 28.98%, Welch-corrected unpaired *t*-test, *p* = 0.22, *n* = 8), IF (81.82 ± 22.88%, unpaired Student’s *t*-test, *p* = 0.50, *n* = 7), IPN (106.3 ± 25.00%, unpaired Student’s *t*-test, *p* = 0.84, *n* = 7), RL (91.27 ± 21.34%, Welsh-corrected unpaired *t*-test, *p* = 0.69, *n* = 7), CLI (80.99 ± 30.01%, Welch-corrected unpaired *t*-test, *p* = 0.54, *n* = 7), RPO (89.85 ± 23.73%, Welch-corrected unpaired *t*-test, *p* = 0.68, *n* = 6), RO (78.84 ± 9.81%, Welch-corrected unpaired *t*-test, *p* = 0.09, *n* = 5), RM (133.00 ± 26.36%, Welch-corrected unpaired *t*-test, *p* = 0.25, *n* = 7), and RPA (144.3 ± 36.00%, Welch-corrected unpaired *t*-test, *p* = 0.28, *n* = 5), were comparable to *wt*.

### Cell Death Analysis in *Tgfβ2^*flox/flox*^:Krox20^*cre/+*^* Animals

**FIGURE 6:**
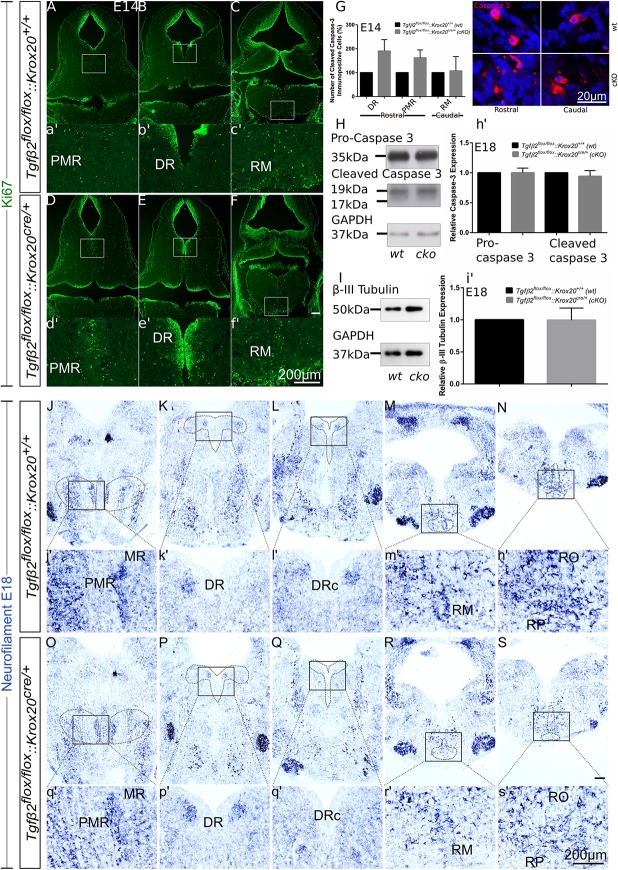
Cell death analysis in hindbrain of *Tgfβ2^*flox/flox*^:Krox20^*cre/+*^* during embryonic development. **(A–F)** Immunofluorescence for the proliferation marker Ki67 in hindbrain coronal fixed cryosections from WT and *Tgfβ2^*flox/flox*^:Krox20^*cre/+*^* at embryonic day (E) 14 shows increased immunoreactivity in the PMR, DR, or RM. (a′–f′) Represent higher magnification of the respective white-boxed areas in **(A–F)**. **(G)** Counting of cleaved caspase 3 immunopositive cells in *wt* and *cKO* at E14 in the areas DR, PMR, and RM. Not significant, using the unpaired two-tailed Student’s *t*-test *n* = 4. Data are given as mean ± SEM, the mean of *wt* littermate was set to 100. **(H,I)** Immunoblot analysis of homogenates from hindbrain of wild type (*wt*) and *Tgfβ2^*flox/flox*^:Krox20^*cre/+*^ (cKO)* shows protein expression of pro-caspase 3, cleaved caspase 3 and β-III tubulin. **(h′,i′)** Quantification after densitometric analysis of the signal ratio protein of interest: GAPDH, not significant, using the two-tailed unpaired Student’s *t*-test, *n* = 4. 30 μg protein was loaded per lane. **(J–S)** ISH for *Neurofilament* in hindbrain of E18 mice. **(j′–s′)** Represent higher magnification of the respective black-boxed areas in **(J–S)**. DR, dorsal raphe; PMR, paramedian raphe; RM, raphe magnus; RO, raphe obscurus; RPA, raphe pallidus. Scale bar: 200 μm, **(G)**: 20 μm.

Having shown that the number of 5-HT neurons is significantly reduced in *cKO* from E14 onwards (Figures 3, 4), but not in adult animals (Figure 5), we have asked whether the phenotype during development can be attributed to decreased proliferation of progenitor cells. To that end, immunofluorescence for the proliferation marker Ki67 at E14 has been performed (Figures 6A–f′). Strikingly, Ki67 immunofluorescence tended to be even more prominent in *cKO* in the area of PMR (Figures 6A,D), DR (Figures 6B,E) or RM (Figures 6C,F), compared to *wt.* Another scenario could be that the decreased number of 5-HT neurons in *cKO* at E14 is due to neuronal loss. Therefore, immunolabelling for cleaved caspase 3 was performed at E14 (Figure 6G) and quantified. At E14, the number of caspase-3 positive cells was increased but not statistically significant in the DR (190.5 ± 47.68%, Welch-corrected unpaired *t*-test, *p* = 0.15, *n* = 4) and PMR (162.5 ± 32.7%; Welch-corrected unpaired *t*-test, *p* = 0.15), compared to the *wt*. Similarly, changes in the number of cleaved caspase 3 in the area of RM (107.5 ± 59.75%; Mann–Whitney *U* = 4, *p* = 0.31) were comparable between *wt* and *cKO*.

Figure 6H illustrates western blot analysis in hindbrain homogenates from *wt* and *cKO* at E18. Pro-caspase 3 was expressed on both *wt* and *cKO* hindbrain, represented by a ∼35 kDa band, whose intensity was comparable between *wt* and *cKO* (1.00 ± 0.07-fold, Welch corrected unpaired *t*-test, *p* = 0.99, *n* = 4, Figure 6h′). Cleaved caspase 3 protein abundance was documented as a strong ∼19 kDa band and a much weaker ∼17 kDa band in both *wt* and *cKO*. Similarly to the data obtained for the pro-caspase 3, no statistical significant differences were assessed between *wt* and *cKO* (0.94 ± 0.09 fold, Welch-corrected unpaired *t*-test, *p* = 0.58, *n* = 4). Consistent with these data, β-III-tubulin expression (∼50 kDa band in Figure 6I) in hindbrain at E18 was similar between *wt* and *cKO* (0.99 ± 0.09 fold, Welch-corrected unpaired *t*-test, *p* = 0.96, Figure 6i′) as well.

Putative changes in neuron abundance at E18 in *wt* and *cKO* has been additionally tested by qualitative ISH for *Neurofilament* (Figures 6J–S). In the areas of MR and PMR (Figures 6J,O and higher magnification of the black-boxed area in 6j′,o′), DRc (Figures 6L,Q and higher magnification of the black-boxed area in 6l′,q′), RM (Figures 6M,R and higher magnification of the black-boxed area in 6m′,r’), RO and RP (Figures 6N,S and higher magnification of the black-boxed area in 6n′,s′) Neurofilament expression in these 5-HT subgroups appeared decreased in *cKO*, compared to *wt.* In contrast, in DR (Figures 6K,P and higher magnification of the black-boxed area in 6k′,p′) no differences between *wt* and *cKO* could be observed.

### Neurochemical Analysis of Hindbrain, Cortex and Hippocampus of *Tgfβ2^*flox/flox*^:Krox20^*cre/+*^* Animals

**FIGURE 7:**
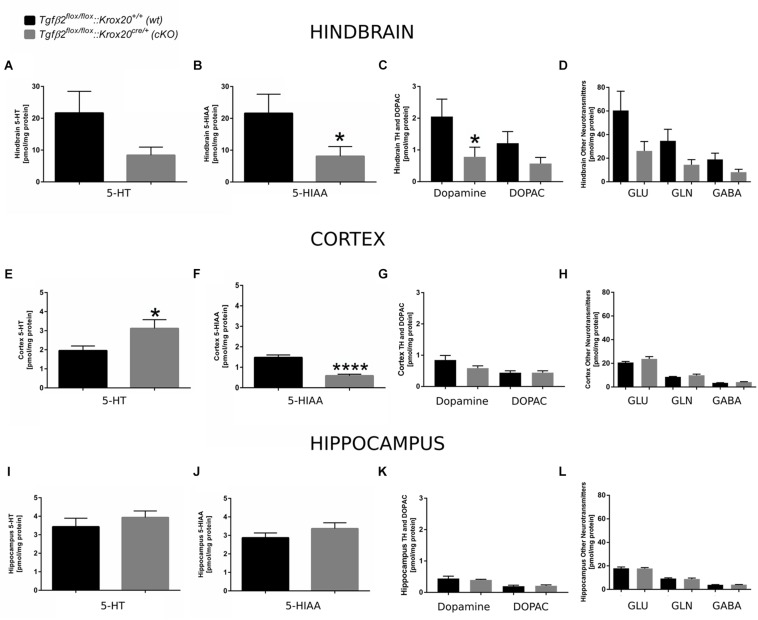
Neurochemical analysis of hindbrain, cortex, and hippocampus of *Tgf*β*2^*flox/flox*^:Krox20^+/+^ (wt) and Tgf*β*2^*flox/flox*^:Krox20^*cre/+*^(cKO)* adult mice. Tissue concentration of 5-HT, 5-HIAA, Dopamine and DOPAC, and glutamate (Glu), Glycine (Gln), and GABA in homogenates of hindbrain **(A–D)**, cortex **(E–H)**, and hippocampus **(I–L)** of *Tgf*β*2^*flox/flox*^:Krox20^+/+^* (*wt, n* = 10–12) and *Tgf*β*2^*flox/flox*^:Krox20^*cre/+*^* (*cKO, n* = 11) adult (3 month old) mice. Data are given as mean ± SEM. ^∗^*p* < 0.05, ^∗∗∗∗^*p* < 0.0001 using the Mann–Whitney test.

Since 5-HT immunoreactive neurons were increased (although not significant) in several raphe nuclei in adult *cKO* animals, we have performed a neurotransmitter screen and quantitated 5-HT and 5-HIAA levels in hindbrain (Figures 7A,B), cortex (whole forebrain; Figures 7E,F), and hippocampal tissue (Figures 7I,J) using HPLC in *wt* and *cKO* at 3 months of age. Moreover, levels of other neurotransmitters and their metabolites, such as dopamine and DOPAC (Figures 7C,G,K), glutamate (Glu), glycine (Gln), and GABA (Figures 7D,H,L) were also determined. Conditional deletion of *Tgf-β2* in rhombomeres 3 and 5 resulted to decreased -but statistically not significant different- of 5-HT levels in the hindbrain of *cKO* (Figure 7A, 8.40 ± 2.52 pmol/mg protein), compared to *wt* (21.65 ± 6.8 pmol/mg protein; Mann–Whitney *U* = 33, *p* = 0.13, 10–12 animals/genotype), together with a significant decrease in 5-HIAA, the main metabolite of 5-HT (Figure 7B, 8.11 ± 3.00 pmol/mg protein and 21.62 ± 5.95 pmol/mg protein for *cKO* and *wt*, respectively; Mann–Whitney *U* = 23, ^∗^*p* = 0.02, 10–12 animals/genotype) compared to *wt*. Similarly, in forebrain, 5-HIAA levels were also significantly decreased (Figure 7F, 0.58 ± 0.07 and 1.47 ± 0.12 pmol/mg for *cKO* and *wt*, respectively, Mann–Whitney *U* = 2, ^∗∗∗∗^*p* < 0.0001), accompanied by a significant increase of 5-HT (Figure 7E, 3.11 ± 0.45 pmol/mg protein, and 1.95 ± 0.23 pmol/mg protein for *cKO* and wt, respectively, Mann–Whitney *U* = 30, ^∗^*p* = 0.04). In the hippocampus both 5-HT (Figure 7I) and 5-HIAA (Figure 7J) levels were comparable between *wt* and *cKO* (Mann–Whitney *U* = 48, *p* = 0.28 and Mann–Whitney *U* = 50, *p* = 0.34 for 5-HT and 5-HIAA, respectively). The levels of dopamine in hindbrain of *cKO* were also significantly decreased, compared to *wt* (Figure 7C; Mann–Whitney *U* = 23, ^∗^*p* = 0.02, 10–11 animals/genotype), whereas all other neurotransmitters and metabolites examined showed no significant differences between *wt* and *cKO* (for DOPAC: Mann–Whitney *U* = 37, *p* = 0.22 (Figure 7C), Glu: Mann–Whitney *U* = 37, *p* = 022, Gln: Mann–Whitney *U* = 35, *p* = 0.17, and GABA: Mann–Whitney *U* = 36, *p* = 0.19; Figure 7D). In cortex and hippocampus, no significant differences for dopamine (Mann–Whitney *U* = 46, *p* = 0.37 and Mann–Whitney *U* = 63, *p* = 0.88, for cortex (Figure 7G) and hippocampus (Figure 7K), respectively), DOPAC (Figures 7G,K) (Mann–Whitney *U* = 52, *p* = 0.86 and *U* = 59, *p* = 0.69, for cortex and hippocampus, respectively), Glu (Figure 7H) Mann–Whitney *U* = 51, *p* = 0.37 and Mann–Whitney *U* = 55, *p* = 0.77, for cortex and hippocampus, respectively), Gln (Figure 7H; Mann–Whitney *U* = 46, *p* = 0.36 and Mann–Whitney *U* = 65, *p* = 0.97, for cortex and hippocampus, respectively), and GABA (Figure 7H; Mann–Whitney *U* = 42, *p* = 0.38 and Mann–Whitney *U* = 52, *p* = 0.86) could be determined between *wt* and *cKO*.

### *Tgfβ2^*flox/flox*^:Krox20^*cre/+*^* Mice Behaviour

**FIGURE 8:**
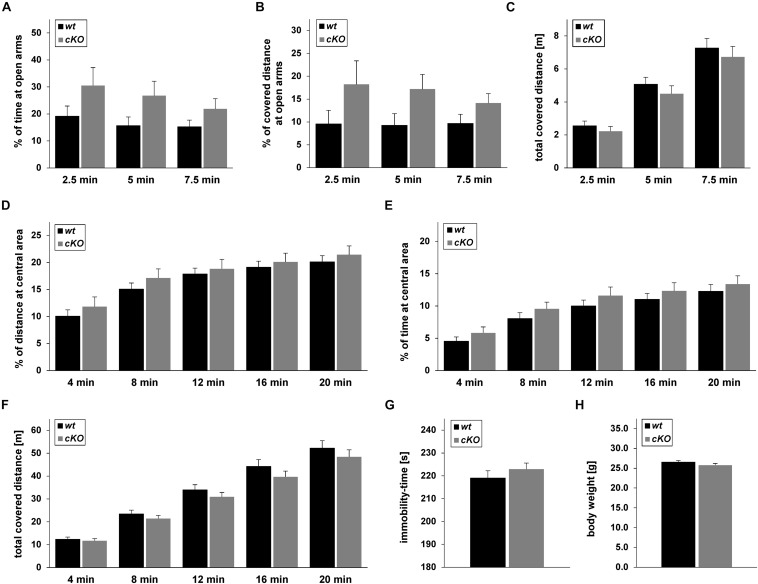
Behaviour of *Tgf*β*2^*flox/flox*^:Krox20^+/+^* (*wt*) and *Tgf*β*2^*flox/flox*^:Krox20^*cre/+*^* (*cKO*) mice. Exploratory behaviour and spontaneous activity of *wt* (*n* = 16) and *cKO* (*n* = 17) mice was recorded for 7.5 min in an elevated plus maze **(A–C)** and 20 min in an open field test **(D–F)**. Percentage of time spent on **(A)** and percentage of covered distance at **(B)** open arms for the first 2.5, 5, and 7.5 min of the elevated plus maze experiment. **(C)** Total covered distance for the same periods. Spontaneous activity of *wt* (*n* = 18) and *cKO* (*n* = 17) mice was recorded for 20 min in the open field arena. Evaluation of data was subdivided in time slots, each one 4 min longer than the previous one. Percentage of time spent **(D)** and percentage of covered distance **(E)** at the inner area of the open field. **(F)** Total distance covered during the habitation period in the open field. **(G)** Immobility-time of *wt* (*n* = 15) and *cKO* (*n* = 17) animals in the forced swim test (FST). **(H)** Body weight of adult (12 weeks) *wt* (*n* = 27) and *cKO* (*n* = 29) mice. Data were analysed using two-tailed Student’s *t*-test. Data are given as mean ± SEM.

Conditional knockouts *(Tgfβ2^*flox/flox*^:Krox20^*cre/+*^*) were born at normal Mendelian ratios and did not show either at birth or during adulthood any noticeable difference from *wt (Tgfβ2^*flox/flox*^:Krox20^+/+^*) or heterozygotes *(Tgfβ2^*flox/+*^:Krox20^+/+^*) mice. Three-month-old animals showed comparable body weight (Figure 8H). *cKO* mice did not reveal any behavioural abnormality with respect to motor activity, feeding, or nest building-behaviour. Similarly, aggressiveness, poor grooming, or any accumulation of injuries was not observed.

Based on the results from the neurochemical screen (Figure 7) we next tested for differences in the behavioural performance between *wt* and *cKO*. Anxiety-like and explorative behaviour was tested on the elevated plus maze (Figures 8A–C). No difference between *wt* and *cKO* in either time spent at open arms (Figure 8A; *p* = 0.17, *p* = 0.23 and *p* = 0.17 after 2.5, 5.0 and 7.5 min, respectively, using the two-tailed unpaired Student’s *t*-test), covered distance at open arms (Figure 8B; *p* = 0.26, *p* = 0.07, and *p* = 0.14, after 2.5, 5.0 and 7.5 min, respectively), as well as of total covered distance after defined time periods (Figure 8C; *p* = 0.43, *p* = 0.39, and *p* = 0.45) was measured in this anxiety test. Similarly, in the open field (Figures 8D–F), data analysis demonstrated that *cKO* showed no differences either in the distance at central area after 4 min (*p* = 0.42), 8 min (*p* = 0.33), 12 min (*p* = 0.66), 16 min (*p* = 0.62) and 20 min (*p* = 0.51) or in the time at central area (*p* = 0.27, *p* = 0.23, *p* = 0.33, *p* = 0.42, *p* = 0.51 after 4, 8, 12, 16, and 20 min, respectively) compared to *wt* (Figures 8D,E). Analysis of total covered distance in the open field (*p* = 0.65, *p* = 0.37, *p* = 0.32, *p* = 0.27 and *p* = 0.41 after 4, 8, 12, 16, and 20 min, respectively) of *wt* and *cKO* animals revealed no significant differences in spontaneous activity (Figure 8F). Thus, both tests indicated a comparable level of anxiety and explorative behaviour for both genotypes. In addition, performance of *cKO* mice during a FST (Figure 8G) was comparable to *wt* littermates (*p* = 0.37).

## Discussion

### Conditional Deletion of *Tgf-β2* Using Cre:Lox

TGF-βs are multifunctional molecules involved in crucial biological processes and established molecular players in the development and maintenance of the nervous system (Ten Dijke and Arthur, 2007; Krieglstein et al., 2011). The three mammalian TGF-β isoforms, i.e., TGF-β1, TGF-β2 and TGF-β3, are encoded by different genes located on different chromosomes (Massague, 1990; Roberts and Sporn, 1990). Mice with constitutive deletion of either isoform or of type II TGF-β receptor reveal distinct phenotypes: mice deficient in the type II TGF-β receptor die at E10.5 (Oshima et al., 1996), *Tgf-β2^–/–^* (Sanford et al., 1997) and *Tgf-β3^–/–^* (Proetzel et al., 1995) die at birth, whereas *Tgf-β1* null mutants die at about 3 weeks of age (Shull et al., 1992). Although all isoforms are expressed in the central nervous system (CNS) under physiological and/or pathophysiological conditions and TGF-β2 is expressed in an overlapping fashion to TGF-β3 during brain development, TGF-β2 is apparently more potent than TGF-β3 during development of neuronal populations (Rahhal et al., 2004; Roussa et al., 2006). Due to lethality of TGF-β null mutants, generation of conditional knockout mice lines is necessary to elucidate the biological significance of each isoform. Mice with a conditional allele for type II *Tgf-β* receptor (Chytil et al., 2002), and *Tgf-β3* (Doetschman et al., 2012) genes are available. Using the targeting strategy shown in Figure 1, *Tgf-β2* floxed mice were generated. Newborn *Tgf-β2^*flox/flox*^* mice were viable, adult *cKO* animals fertile, and indistinguishable from their WT littermates. The design of the *Tgf-β2* targeted allele permits removal of LoxP-flanked *Tgf-β2* genomic sequence by mating to a Cre line. Here, we have made use of cell type specific deletion of TGF-β2 ligand, using the Cre-loxP-system and deleted *Tgf-*β*2* from Krox20-expressing cells. Lineage analysis of the serotonergic system (Jensen et al., 2008; Alonso et al., 2013) has revealed that r3 together with r1 and r2 contributes to the generation of a number of 5-HT neurons within the B9/B8/B5 (MR), whereas B3/B1 (RM) developmentally derives from r5 and r6. Since Krox20 is exclusively expressed in r3 and r5 in the developing brain, in the conditional knockout mice used in the present study, the ligand TGF-β2 was deleted from some MR, PMR, and RM progenitors and from progenitors of other non-serotonergic neuronal populations.

### Temporal Patterns of Serotonergic Neuron Response to TGF-β2

TGF-β2 and TGF-β3 ligands together with type II TGF-β receptor are expressed in mouse hindbrain floor from E12.5 onwards (Flanders et al., 1991; Galter and Unsicker, 1999, 2000). Based on this observation the hypothesis has been formulated that TGF-βs might be inductive molecular cues for the development and survival of hindbrain 5-HT neurons, as already shown for the developmentally related midbrain dopaminergic neurons (Roussa et al., 2006; Chleilat et al., 2018). The results of the present study show that loss of TGF-β2 from r3/r5 resulted to significantly decreased the number of caudal hindbrain 5-HT neurons at E14 (Figure 3) but not at E12 (Figure 2). At E12 however, *Pet1* expression area was reduced in *cKO*, presumably first hint for the phenotype observed at E14. In a previous study we have shown that *Tgf-*β*2*^–/^*^–^* mice reveal significant decreased number of rostral hindbrain 5-HT neurons as early as E12 and a selective growth factor dependency of PMR neurons at E18 (Chleilat et al., 2018). Moreover, conditional deletion of TGF-β signalling in r1-expressing cells has been shown selective dependency of caudal DR neuron development on TGF-β signalling (Chleilat et al., 2018). Since caudal hindbrain serotonergic subpopulations develop later, as compared to rostral 5-HT subpopulations, the “delayed” TGF-β2-dependency of caudal 5-HT neuron development is likely related to a specific developmental stage rather than to distinct subgroups of hindbrain 5-HT neurons (rostral vs. caudal) or a delay in their differentiation, as in the case of lack of TGF-β signalling (Dias et al., 2014). Temporally distinct responses of hindbrain serotonergic neurons to the neurotrophins BDNF and NT-3 as well as to TGF-βs have been previously observed in primary cultures from rat embryonic hindbrain (Galter and Unsicker, 1999, 2000; Galter et al., 1999; Rumajogee et al., 2002). At E18 (Figure 4), the number of 5-HT immunopositive cells was significantly decreased in RM, MR, and PMR serotonergic neurons, and additionally in the caudal part of DR, and in RO in the *cKO*. The reduction of 5-HT neurons from RM, MR, and PMR can be easily explained by the contribution of r3 and r5 in their development and this observation impressively highlights the importance of TGF-β2 in the development of these serotonergic subpopulations: even partial TGF-β2 deficiency, i.e., only in r3, during development is apparently sufficient to cause a phenotype in MR, PMR, and RM. With regard to the different rhombomeric origin of DRc (from r1), at first glance these results appear intriguing and contradictory. However, r1-derived components can be further subdivided into midbrain, isthmic, and r1 parts (Alonso et al., 2013), observations that provide a clear cut of the DR into a rostral and caudal part. The later shares similarities with MR, is even viewed as a dorsal extension of the MR (Commons, 2015), and is distinct from the rostral DR. Besides developmentally common origin, DRc and MR also share similar connections and are associated with response to stressful or adverse circumstances (Konno et al., 2007; Sperling and Commons, 2011). Our results extend these observations and show comparable response and dependency of developing DRc and MR serotonergic neurons on TGF-β2. What is not clear is the underlying basis for the reduced number of 5-HT neurons in the r6-derived RO in cKO.

The reduced number of 5-HT immunopositive cells in DRc, MR and RM of cKO could be either a result of reduced proliferation of serotonergic progenitors at earlier developmental stages or of increased cell death or of loss of neurotransmitter phenotype. Our results show reduced expression of the neural marker Neurofilament at E18 in *cKO* (Figures 6J–S) supporting a neuronal loss rather than a neurotransmitter loss as the cause for the serotonergic phenotype observed. Moreover, while proliferation of progenitors at E14 was increased in *cKO*, compared to *wt* (Figures 6A–f′), the number of cleaved-caspase 3-positive cells was increased in DR and PMR area (Figure 6G). Due to the considerable biological variability between animals of the same genotype, reflected by the variability of absolute numbers of immunopositive neurons, the differences between *wt* and *cKO* were not statistically significant. Based on these observations the phenotypes obtained are also consistent with a developmental delay of caudal 5-HT positive neurons. Western blot analysis showed no differences on cleaved caspase 3 protein expression between *cKO* and *wt* as well (Figure 6H), a result that can be explained by the use of hindbrain tissue deriving from more rhombomeres than r3 and r5.

The temporal dynamics of hindbrain serotonergic neurons on TGF-β2 became evident in adult stages. Surprisingly, the serotonergic phenotype observed in embryonic stages was restored in 3-month-old animals, and with regard to DR neurons the phenotype was even overshot (Figure 5). These data imply that TGF-β2 is critically involved in the development and specification of hindbrain serotonergic neurons and other endogenously expressed TGF-β isoforms, i.e., TGF-β3, cannot compensate for TGF-β2 loss. In contrast, in adult hindbrain serotonergic neurons chronic compensatory mechanisms may contribute to the restored and overshot phenotype and need to be considered.

### Biological Significance of TGF-β2 Depletion in r3/r5 Neurons: Evidence for Impaired Neurotransmitter Synthesis and Turnover and Serotonin Accumulation in the Forebrain

TGF-β signalling pathways are established molecular players involved in the modulation of both excitatory and inhibitory synaptic transmission in the adult mammalian brain (reviewed in Krieglstein et al., 2011). Based on this background together with the observed serotonergic phenotype in adult *wt* and *cKO*, we have determined the neurochemical profile of *cKO* and *wt* in the hindbrain, cortex, and hippocampus (Figure 7). 5-HT levels in hindbrain of *cKO* were considerably decreased, although not significant, and correlated with 5-HIAA compared to *wt*, with comparable or even increased number of 5-HT synthesising neurons. These data strongly suggest decreased 5-HT synthesis and decreased metabolism in the brainstem. It was therefore surprising that a significant increase in 5-HT and a significant decrease in 5-HIAA -ultimately resulted to a significant increased 5-HT:5-HIAA-ratio- in the cortex of *cKO*, compared to *wt* animals. These results may imply an increased transport to or release of the neurotransmitter from nerve terminals in the cortex together with reduced 5-HT metabolism in the cortex of *cKO*. Since 5-HIAA levels are however significantly decreased in the cortex in *cKO*, compared to *wt*, the most possible scenario could be that 5-HT merely accumulates in the forebrain. Such accumulation could reflect compensatory mechanisms to maintain forebrain serotonin levels despite reduced 5-HT available from the hindbrain. TGF-β2 is secreted and acts at both autocrine and paracrine mode. Though TGF-β2 is depleted from certain 5-HT-producing cells only, its loss during development and adulthood might have chronically influenced function and neurotransmitter release of adjacent serotonergic neurons.

With regard to other neurotransmitters, a significant decrease in the level of dopamine could be observed in hindbrain in *cKO*, a result of either reduced local dopamine synthesis in dopamine-producing neurons residing in the hindbrain or to reduced number of dopaminergic fibers that innervate the rostral hindbrain. Thus, TGF-β2 might be directly or indirectly potent to modulate not only serotonin but other transmitter synthesis and metabolism as well. In a previous study, functional analysis of the preBötzinger complex of *Tgf-β2^–/–^* at E18.5 revealed that loss of TGF-β2 mainly impairs the presynaptic component of both the inhibitory and excitatory synaptic transmission (Heupel et al., 2008). As component of the brainstem respiratory rhythm-generating network, the preBötC complex is at E18.5 functionally more mature than other neuronal networks of the brain (Smith et al., 1991; Hübner et al., 2001; Koizumi et al., 2013). TGF-β2 has also been shown to regulate presynaptic quantal size at the neuromuscular junction (Fong et al., 2010). In the context of the present work, the questions whether and if yes, how altered neurotransmitter levels in hindbrain and forebrain of *cKO* might translate into altered electrophysiological properties of the respective neurons and/or altered plasticity need to be addressed by functional studies.

### Behavioural Outcome

The putative physiological significance of increased DR serotonergic neurons and altered neurochemical profile of *cKO* animals prompted us to investigate whether it is associated with altered behavioural performance of *cKO* animals. The link between 5-HT levels and neuropsychiatric disorders has been firmly established. DR and MR neurons are the main sources for serotonergic innervation of the forebrain and therefore are considered most relevant in modulating behaviour. Using optogenetics or pharmacogenetic activation of selective 5-HT neurons many studies have uncovered important insights on the contribution of individual raphe nuclei to distinct behaviours (Demarque and Spitzer, 2010; Teissier et al., 2015; Niederkofler et al., 2016). Using the anxiety-related elevated plus maze and open field tests no differences in the behavioural outcome between the genotypes could be observed, demonstrating unaltered anxiety levels in *cKO*. We applied the FST to uncover possible depressive-like behaviour, and again, no differences were detected between the genotypes. These results however, do not exclude an altered response of mutant mice to external stressors. Moreover, since serotonergic neurons of RO innervate the pre-Bötzinger complex and have also been shown to activate breathing frequency (Depuy et al., 2011), investigating respiratory functions and the respiratory chemoreflex might be promising tests that need to be addressed.

In summary, the present work demonstrates differential spatial and temporal responsiveness of developing and adult serotonergic neurons on TGF-β2. Whether the observed effects during development of the serotonergic system are isoform-specific or not, investigation regarding the functional impact of other isoforms in parallel would be necessary. The results also indicate TGF-β2 being directly or indirectly potent to modulate neurotransmitter synthesis and metabolism. Moreover, we introduce a novel genetic tool, the floxed TGF-β2 mouse line, suitable for analysing the *in vivo* functions of TGF-β2 during development and in adulthood in many organs.

## Data Availability Statement

All datasets generated for this study are included in the manuscript/supplementary files.

## Ethics Statement

All protocols were carried out in accordance with the German ethical guidelines for laboratory animals and approved by the Institutional Animal Care and Use Committee of the City of Freiburg and the University of Freiburg (authorizations: G11/56, G17/008, and X-16/07S).

## Author Contributions

EC performed the immunohistochemistry analysis and quantification, made genotyping of the lines, and contributed to the analysis/assembly of data for all figures. RM and NK performed and analysed the behavioural studies. RS performed and analysed the neurochemical analysis. KK contributed to the concept of the manuscript and management of the project. ER was in charge for the conception and management of the project and wrote the manuscript. All authors approved the submitted manuscript.

## Conflict of Interest

The authors declare that the research was conducted in the absence of any commercial or financial relationships that could be construed as a potential conflict of interest.

## Footnotes

1 http://www.ensembl.org/Mus_musculus/

## Abbreviations

cKO: conditional knockout
CLI: central linear nucleus
CSl, superior central nucleus raphe: lateral part
CSm, superior central nucleus raphe: medial part
DOPAC: 3,4-dihydroxyphenylacetic acid
DR: dorsal raphe nucleus
Egr2/Krox20: early growth response-2
EGFP: enhanced green fluorescent protein
E#: embryonic day
FST: forced swim test
GABA: gamma-aminobutyric acid
Glu: glutamate
Gln: glycine
5-HIAA: 5-hydroxyindoleacetic acid
5-HT: 5-hydroxytryptamine or serotonin
HPLC: high performance liquid chromatography
IF: interfascicular nucleus alpha
IPN: interpenducular nucleus
ISH: *in situ* hybridization
loxDM: double mutant
MR: median raphe
PMR: paramedian raphe
preBötC: pre-Bötzinger complex
r3/r5: rhombomere 3/5
RL: rostral linear nucleus
RM: raphe magnus
RO: raphe obscurus/oralis
RPA: raphe pallidus
RPO: pontine nucleus raphe
TGF- β 2: transforming growth factor beta 2
TH: tyrosine hydroxylase or dopamine
WT: wild type

## References

[B1] Alonso A. Merchan P. Sandoval J. E. Sanchez-Arrones L. Garcia-Cazorla A. Artuch R. (2013). Development of the serotonergic cells in murine raphe nuclei and their relations to rhombomeric domains. *Brain Struct. Funct.* 218 1229–1277. 10.1007/s00429-012-0456-8 23052546 PMC3748323

[B2] Bang S. J. Jensen P. Dymecki S. M. Commons K. G. (2012). Projections and interconnections of genetically defined serotonin neurons in mice. *Eur. J. Neurosci.* 35 85–96. 10.1111/j.1460-9568.2011.07936.x 22151329 PMC3268345

[B3] Brust R. D. Corcoran A. E. Richerson G. B. Nattie E. Dymecki S. M. (2014). Functional and developmental identification of a molecular subtype of brain serotonergic neuron specialized to regulate breathing dynamics. *Cell Rep.* 9 2152–2165. 10.1016/j.celrep.2014.11.027 25497093 PMC4351711

[B4] Can A. Dao D. T. Arad M. Terrillion C. E. Piantadosi S. C. Gould T. D. (2012). The mouse forced swim test. *J. Vis. Exp.* 59:e3638. 10.3791/3638 22314943 PMC3353513

[B5] Chleilat E. Skatulla L. Rahhal B. Hussein M. T. Feuerstein M. Krieglstein K. (2018). TGF-β signaling regulates development of midbrain dopaminergic and hindbrain serotonergic neuron subgroups. *Neuroscience* 381 124–137. 10.1016/j.neuroscience.2018.04.019 29689292

[B6] Chytil A. Magnuson M. A. Wright C. V. Moses H. L. (2002). Conditional inactivation of the TGF-beta type II receptor using Cre:lox. *Genesis* 32 73–75. 10.1002/gene.10046 11857781

[B7] Commons K. G. (2015). Two major network domains in the dorsal raphe nucleus. *J. Comp. Neurol.* 523 1488–1504. 10.1002/cne.23748 25652113 PMC4439335

[B8] Demarque M. Spitzer N. C. (2010). Activity-dependent expression of Lmx1b regulates specification of serotonergic neurons modulating swimming behavior. *Neuron* 67 321–334. 10.1016/j.neuron.2010.06.006 20670838 PMC2913149

[B9] Depuy S. D. Kanbar R. Coates M. B. Stornetta R. L. Guyenet P. G. (2011). Control of breathing by raphe obscurus serotonergic neurons in mice. *J. Neurosci.* 31 1981–1990. 10.1523/JNEUROSCI.4639-10.2011 21307236 PMC3071248

[B10] Dias J. M. Alekseenko Z. Applequist J. M. Ericson J. (2014). TGFβ signalling regulates temporal neurogenesis and potency of neural stem cells in the CNS. *Neuron* 84 927–939. 10.1016/j.neuron.2014.10.033 25467979

[B11] Doetschman T. Georgieva T. Li H. Reed T. D. Grisham C. Friel J. (2012). Generation of mice with conditional allele for the transforming growth factor beta 3 gene. *Genesis* 50 59–66. 10.1002/dvg.20789 22223248 PMC3850393

[B12] Ernsberger U. Patzke H. Tissier-Seta J. P. Reh T. Goridis C. Rohrer H. (1995). The expression of tyrosine hydroxylase and the transcription factors cPhox-2 and cash-1: evidence for distinct inductive steps in the differentiation of chick sympathetic precursor cells. *Mech. Dev.* 52 125–136. 10.1016/0925-4773(95)00396-i 7577670

[B13] Flanders K. C. Lüdecke G. Engels S. Cissel D. S. Roberts A. B. Kondaiah P. (1991). Localization and action of transforming growth factor-betas in the embryonic nervous system. *Development* 113 183–191. 1764993 10.1242/dev.113.1.183

[B14] Fong S. W. McLennan I. S. McIntyre A. Reid J. Shennan K. I. Bewick G. S. (2010). TGF-beta2 alters the characteristics of the neuromuscular junction by regulating presenaptic quantal size. *Proc. Natl. Acad. Sci. U.S.A.* 107 13515–13519. 10.1073/pnas.1001695107 20624974 PMC2922142

[B15] Galter D. Böttner M. Unsicker K. (1999). Developmental regulation of the serotonergic transmitter phenotype in rostral and caudal raphe nucleus by transforming growth factor betas. *J. Neurosci. Res.* 56 531–538. 10.1002/(sici)1097-4547(19990601)56:5<531::aid-jnr8>3.0.co;2-o 10369219

[B16] Galter D. Unsicker K. (1999). Regulation of the transmitter phenotype of rostral and caudal groups of cultured serotonergic raphe neurons. *Neuroscience* 88 549–559. 10.1016/s0306-4522(98)00224-3 10197774

[B17] Galter D. Unsicker K. (2000). Brain-derived neurotrophic factor and trk-B are essential for cAMP-mediated induction of the serotonergic neuronal phenotype. *J. Neurosci. Res.* 61 295–301. 10.1002/1097-4547(20000801)61:3<295::aid-jnr7>3.3.co;2-w 10900076

[B18] Garratt A. N. Voiculescu O. Topilko P. Charnay P. Birchmeier C. (2000). A dual role of erbB2 in myelination and in expansion of the schwann cell precursor pool. *J. Cell Biol.* 148 1035–1046. 10.1083/jcb.148.5.1035 10704452 PMC2174554

[B19] Haugas M. Tikker L. Achim K. Salminen M. Partanen J. (2016). Gata2 and Gata3 regulate the differentiation of serotonergic and glutamatergic neuron subtypes of the dorsal raphe. *Development* 143 4495–4508. 10.1242/dev.136614 27789623

[B20] Hendricks T. Francis N. Fyodorov D. Deneris E. S. (1999). The ETS domain factor of Pet-1 is an early and precise marker of central serotonin neurons and interacts with a conserved element in serotonergic genes. *J. Neurosci.* 19 10348–10356. 10.1523/jneurosci.19-23-10348.1999 10575032 PMC6782418

[B21] Heupel K. Sargsyan V. Plomp J. J. Rickmann M. Varoqueaux F. Zhang W. (2008). Loss of transforming growth factor-beta 2 leads to impairment of central synapse function. *Neural Dev.* 3:25. 10.1186/1749-8104-3-25 18854036 PMC2576228

[B22] Huber K. Bruhl B. Guillemot F. Olson E. N. Ernsberger U. Unsicker K. (2002). Development of chromaffin cells depends on MASH1 function. *Development* 129 4729–4738. 12361965 10.1242/dev.129.20.4729

[B23] Hübner C. A. Stein V. Hermans-Borgmeyer I. Meyer T. Ballanyi K. Jentsch T. J. (2001). Disruption of KCC2 reveals an essential role of K-Cl cotransport already in early synaptic inhibition. *Neuron* 30 515–524. 10.1016/s0896-6273(01)00297-5 11395011

[B24] Jensen P. Farago A. F. Awartamani R. B. Scott M. M. Deneris E. S. Dymecki S. M. (2008). Redefining the serotonergic system by genetic lineage. *Nat. Neurosci.* 11 417–419. 10.1038/nn2050 18344997 PMC2897136

[B25] Koizumi H. Koshiya N. Chia X. J. Cao F. Nugent J. Zhang R. (2013). Structural-functional properties of identified excitatory and inhibitory interneurons within Pre-Bötzinger complex respiratory microcircuits. *J. Neurosci.* 33 2994–3009. 10.1523/jneurosci.4427-12.2013 23407957 PMC3707631

[B26] Konno K. Matsumoto M. Togashi H. Yamaguchi T. Izumi T. Watanabe M. (2007). Early postnatal stress affects the serotonergic function in the median raphe nuclei of adult rats. *Brain Res.* 1172 60–66. 10.1016/j.brainres.2007.05.022 17825274

[B27] Krieglstein K. Zheng F. Unsicker K. Alzheimer C. (2011). More than being protective: functional roles for TGF-β/activin signaling pathways at central synapses. *Trends Neurosci.* 34 421–429. 10.1016/j.tins.2011.06.002 21742388

[B28] Labalette C. Wassef M. A. Desmarquet-Trin Dinh C. Bouboucha Y. X. Le Men J. Charnay P. (2015). Molecular dissection of segment formation in the developing hindbrain. *Development* 142 185–195. 10.1242/dev.109652 25516974

[B29] Mallmann R. T. Elgueta C. Sleman F. Castonguay J. Wilmes T. van den Maagdenberg A. (2013). Albaltion of Cav2.1 voltage-gated Ca2+ channels in mouse forebrain generates multiple cognitive impairments. *PLoS One* 10:e78598. 10.1371/journal.pone.0078598 24205277 PMC3814415

[B30] Massague J. (1990). The transforming growth factor beta family. *Annu. Rev. Cell Biol.* 6 597–641. 2177343 10.1146/annurev.cb.06.110190.003121

[B31] Niederkofler V. Asher T. E. Okaty B. W. Rood B. D. Narayan A. Hwa L. S. (2016). Identification of serotonergic neuronal modules that affect aggressive behavior. *Cell Rep.* 17 1934–1949. 10.1016/j.celrep.2016.10.063 27851959 PMC5156533

[B32] Oberdoerffer P. Otipoby K. L. Maruyama M. Rajewsky K. (2003). Unidirectional cre-mediated genetic inversion in mice using the mutant loxP pair lox66/lox71. *Nucleic Acids Res.* 31:e140. 14602933 10.1093/nar/gng140 PMC275577

[B33] Okaty B. W. Frerer M. E. Rood B. D. Brust R. D. Hennessy M. L. deBairos D. (2015). Multi-scale molecular deconstruction of the serotonin neuron system. *Neuron* 88 774–791. 10.1016/j.neuron.2015.10.007 26549332 PMC4809055

[B34] Oshima M. Oshima H. Taketo M. M. (1996). TGF-beta receptor type II deficiency results in defects of yolk sac hematopoiesis and vasculogenesis. *Dev. Biol.* 179 297–302. 10.1006/dbio.1996.0259 8873772

[B35] Osterberg N. Wiehle M. Oehlke O. Heidrich S. Xu C. Fan C. M. (2011). Sim1 is a novel regulator in the differentiation of mouse dorsal raphe serotonergic neurons. *PLoS One* 6:e19239. 10.1371/journal.pone.0019239 21541283 PMC3082558

[B36] Paxinos G. Watson C. (1998). *The Rat Brain in Stereotaxic Coordinates.* Cambridge, MA: Academic press.

[B37] Proetzel G. Pawlowski S. A. Wiles M. V. Yin M. Boivin G. P. Howles P. N. (1995). Transforming growth factor-beta 3 is required for secondary palate fusion. *Nat. Genet.* 11 409–414. 10.1038/ng1295-409 7493021 PMC3855390

[B38] Rahhal B. Dünker N. Combs S. Krieglstein K. (2004). Isoform-specific role of transforming growth factor-beta2 in the regulation and differentiation of murine adrenal chromaffin cells in vivo. *J. Neurosci. Res.* 78 493–498. 10.1002/jnr.20286 15478122

[B39] Roberts A. B. Sporn M. B. (ed) (1990). “The transforming growth factor betas,” in *Peptide Growth Factors and Their Receptors – Handbook of Experimental Pharmacology*, (Heidelberg: Springer), 419–472.

[B40] Roussa E. Wiehle M. Dünker N. Becker-Catins S. Oehlke O. Krieglstein K. (2006). Transforming growth factor beta is required for differentiation of mouse mesencephalic progenitors into dopaminergic neurons in vitro and in vivo: ectopic induction in dorsal mesencephalon. *Stem Cells* 24 2120–2129. 10.1634/stemcells.2005-0514 16741229

[B41] Rumajogee P. Madeira A. Verge D. Hamon M. Miquel M. C. (2002). Up-regulation of the neuronal serotoninergic phenotype in vitro: BDNF and cAMP share Trk-B-dependent mechanisms. *J. Neurochem.* 83 1525–1528. 10.1046/j.1471-4159.2002.01264.x 12472905

[B42] Sanford L. P. Ormsby I. Gittenberger-de Groot A. C. Sariola H. Friedman R. Boivin G. P. (1997). TGFbeta2 knockout mice have multiple developmental defects that are non-overlapping with other TGFbeta knockout phenotypes. *Development* 124 2659–2670. 9217007 10.1242/dev.124.13.2659 PMC3850286

[B43] Schneider-Maunoury S. Seitanidou T. Charnay P. Lumdsen A. (1997). Segmental and neuronal architecture of the hindbrain of Krox-20 mouse mutants. *Development* 124 1215–1226. 9102308 10.1242/dev.124.6.1215

[B44] Scicchitano M. S. Dalmas D. A. Boyce R. W. Thomas H. C. Frazier K. S. (2009). Protein extraction of formalin-fixed, paraffin-embedded tissue enables proteomic profiles by mass spectrometry. *J. Histochem. Cytochem.* 57 849–860. 10.1369/jhc.2009.953497 19471015 PMC2728129

[B45] Shull M. M. Ormsby I. Kier A. B. Pawlowski S. Diebold R. J. Yin M. (1992). Targeted disruption of the mosue transforming growth factor-beta 1 gene results in multifocal inflammatory disease. *Nature* 359 639–699. 10.1038/359693a0 PMC3889166 1436033

[B46] Smith J. C. Ellenberger H. H. Ballanyi K. Richter D. W. Feldman J. L. (1991). Pre-Bötzinger complex: a brainstem region that may generate respiratory rhythm in mammals. *Science* 254 726–729. 10.1126/science.1683005 1683005 PMC3209964

[B47] Sperling R. Commons K. G. (2011). Shifting topographic activation and 5-HT1A receptor-mediated inhibition of dorsal raphe serotonin neurons produced by nicotine exposure and withdrawal. *Eur. J. Neurosci.* 33 1866–1875. 10.1111/j.1460-9568.2011.07677.x 21501256 PMC3095684

[B48] Teissier A. Chemiakine A. Inbar B. Bagchi S. Ray R. S. Palmiter R. D. (2015). Activity of raphe serotonergic neurons controls emotional behaviors. *Cell Rep.* 13 1965–1976. 10.1016/j.celrep.2015.10.061 26655908 PMC4756479

[B49] Ten Dijke P. Arthur H. M. (2007). Extracellular control of TGFβ signalling in vascular development and disease. *Nat. Rev. Mol. Cell Biol.* 8 857–869. 10.1038/nrm2262 17895899

[B50] Topilko P. Schneider-Maunoury S. Levi G. Baron-van Evercooren A. Chennoufi A. B. Y. Seitanidou T. (1994). Krox-20 controls myelination in the peripheral nervous system. *Nature* 371 796–799. 10.1038/371796a0 7935840

[B51] van Praag H. Kempermann G. Gage F. H. (2000). Neural consequences of environmental enrichment. *Nat. Rev. Neurosci.* 1 191–198. 11257907 10.1038/35044558

[B52] Vengeliene V. Bespalov A. Roßmanith M. Horschitz S. Berger S. Relo A. L. (2017). Towards trans-diagnostic mechanisms in psychiatry: neurobehavioral profile of rats with a loss-of-function point mutation in the dopamine transporter gene. *Dis. Mod. Mech.* 10 451–461. 10.1242/dmm.027623 28167616 PMC5399565

[B53] Voiculescu O. Taillebourg E. Pujades C. Kress C. Buart S. Charnay P. (2001). Hindbrain patterning: krox20 couples segmentation and specification of regional identity. *Development* 128 4967–4978. 11748134 10.1242/dev.128.24.4967

[B54] Whitney M. S. Shemery A. M. Yaw A. M. Donovan L. J. Glass D. Deneris S. (2016). Adult brain serotonin deficiency causes hyperactivity, cicardian disruption, and elimination of Siestas. *J. Neurosci.* 36 9828–9842. 10.1523/JNEUROSCI.1469-16.2016 27656022 PMC5030349

[B55] Wylie C. J. Hendricks T. J. Zhang B. Wang L. Lu P. Leahy P. (2010). Distinct transcriptomes define rostral and caudal serotonin neurons. *J. Neurosci.* 30 670–684. 10.1523/JNEUROSCI.4656-09.2010 20071532 PMC3403750

[B56] Zhang C. Lutz B. (2002). Cre recombinase-mediated inversion using lox66 and lox71: method to introduce conditional point mutations into the CREB-binding protein. *Nucleic Acids Res.* 30:e90. 12202778 10.1093/nar/gnf089 PMC137435

